# Anatomical Variations of the Cystic Artery and Laparoscopic Cholecystectomy: A Persisting Surgical Challenge

**DOI:** 10.7759/cureus.67948

**Published:** 2024-08-27

**Authors:** Miltiadis Perdikakis, Artemis Liapi, Andreas Kiriakopoulos, Dimitrios Schizas, Evangelos Menenakos, Orestis Lyros

**Affiliations:** 1 5th Department of Surgery, Evgenidion Hospital, School of Medicine, National and Kapodistrian University of Athens, Athens, GRC; 2 3rd Department of Surgery, Euroclinic Hospital, Athens, GRC; 3 Department of Radiology, Euroclinic Hospital, Athens, GRC

**Keywords:** surgical case report, arterial variations, cystic artery, middle hepatic artery, laparoscopic cholecystectomy

## Abstract

Although elective laparoscopic cholecystectomy is a common surgical procedure, it can become challenging due to multiple variants of the anatomy of both cystic artery and cystic bile duct. A 52-year-old male with a history of symptomatic cholelithiasis underwent elective laparoscopic cholecystectomy. During preparation of the Calot’s triangle in order to achieve the “critical view of safety”, an uncommon variation of the arterial anatomy was detected. The cystic artery was found to be originating from a robust middle hepatic artery instead of the right hepatic artery. The retrograde manner of cholecystectomy helped the visualization and protection of the middle hepatic artery. This anatomic finding was confirmed per CT done postoperatively. This case constitutes a rare arterial variation, in which the cystic artery arises from the middle hepatic artery, the artery that supplies the hepatic segment IV, which itself constituted a rare variation, since it arose from the anterior branch of the right hepatic artery. This artery could be falsely ligated instead of the real cystic artery. Certain techniques can be used to enhance the surgeon’s ability to distinguish and safely ligate the proper entities. Anatomic knowledge of the possible variations of arterial and bile vessels is crucial for intraoperative recognition. Dissection of the Calot’s triangle and reassurance of the “critical view of safety” are mandatory dissection techniques during laparoscopic cholecystectomy. Additionally, the retrograde manner of cholecystectomy can be of significant help in case of unclear anatomy in order to avoid ligation of uncertain entities during dissection.

## Introduction

Laparoscopic cholecystectomy is a common surgical procedure; however, multiple challenges arise due to the variant anatomy of the blood and bile vessels of the operative field, which can vary from hematomas to major hemorrhages [1]. Herein, we present a case report of a rare variation of the cystic artery (CA), which was identified during elective laparoscopic cholecystectomy. The anatomical structures identified were significantly distinct from the commonly recognized origins of the CA, which usually arises from the right hepatic artery (RHA) [2]. In our case, the CA constituted a branch of a middle hepatic artery (MHA) coursing similarly to the typical CA, on its way to supply the IV hepatic segment. Interestingly, as depicted radiologically, the present MHA arose from the anterior branch of the RHA, a further rare anatomical variation. Given the current case, we concisely discuss the arterial variations of the gallbladder blood supply, emphasizing the importance of anatomical knowledge of possible variations along with sufficient clarification of the anatomy during preparation in order to avoid severe consequences of cholecystectomy. Finally, we discuss two techniques to prevent these consequences: the dissection of Calot’s triangle and the attainment of the “critical view of safety” [3]. Certainly, open cholecystectomy is a useful option in urgent or complex cases, in which the patient's status is not compatible with typical laparoscopic cholecystectomy [4].

## Case presentation

**Figure 1:**
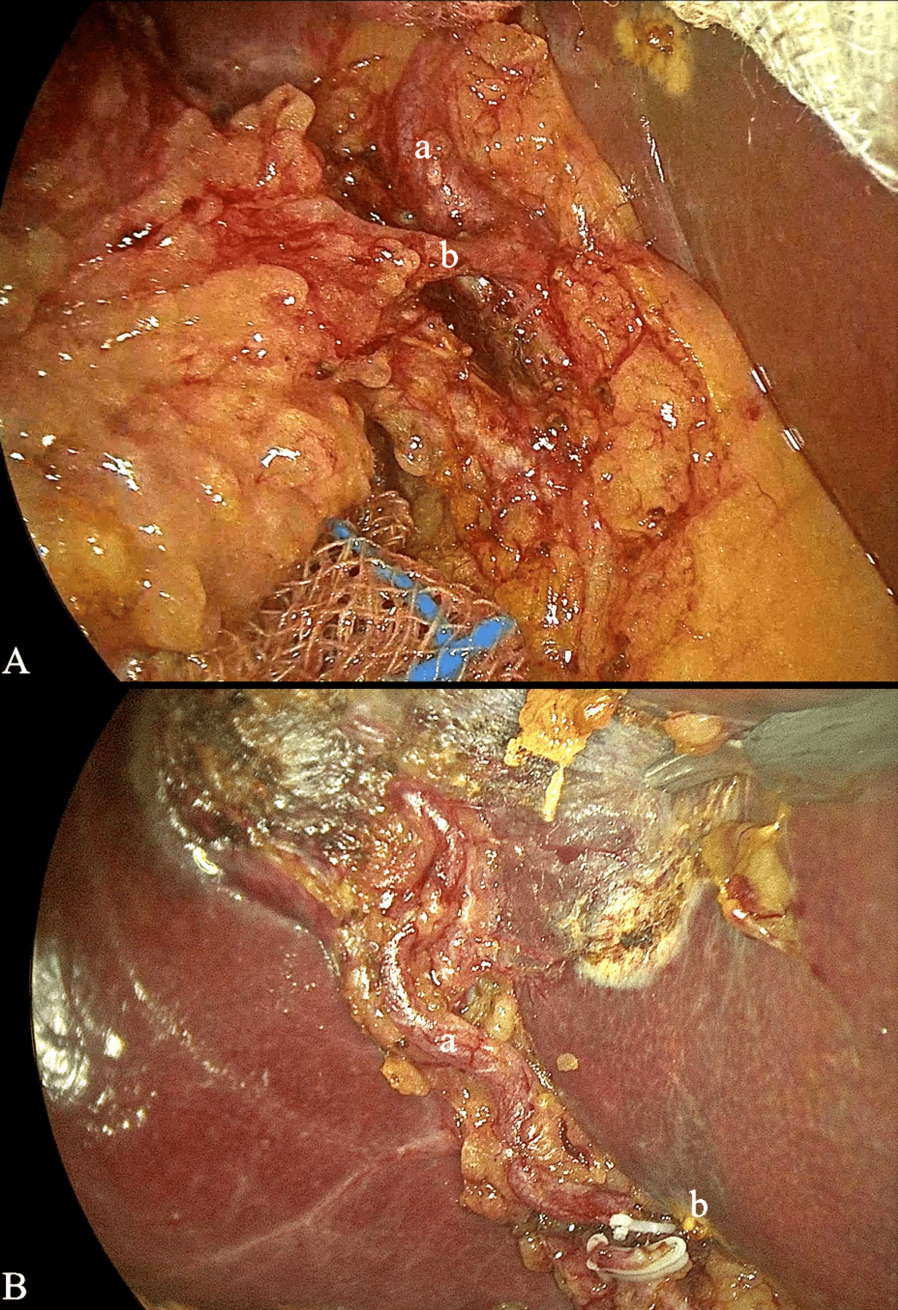
Laparoscopic image of the two arteries (a) Middle hepatic artery, (b) cystic artery (clipped in B)

A 52-year-old male with a history of symptomatic cholelithiasis underwent an elective laparoscopic cholecystectomy. Intraoperatively, during the dissection in an effort to secure the “critical view of safety”, an unusual arterial variation was identified. The CA, which typically originates directly from the RHA, in this case originated from a robust MHA. The MHA demonstrated a course similar to that expected for a common CA and could easily be ligated. However, due to the thorough dissection of the operative field in order to clarify the Calot’s triangle and finally achieve the “critical view of safety”, the middle hepatic artery was identified with a serpentine course providing blood supply to the IV hepatic segment. This artery could easily be falsely ligated in a rushed operation. The identification of the MHA facilitated the identification and final proper ligation of the CA. This unusual course of the MHA artery and the origin of the CA constitute a rare variation (Figure 1).

**Figure 2:**
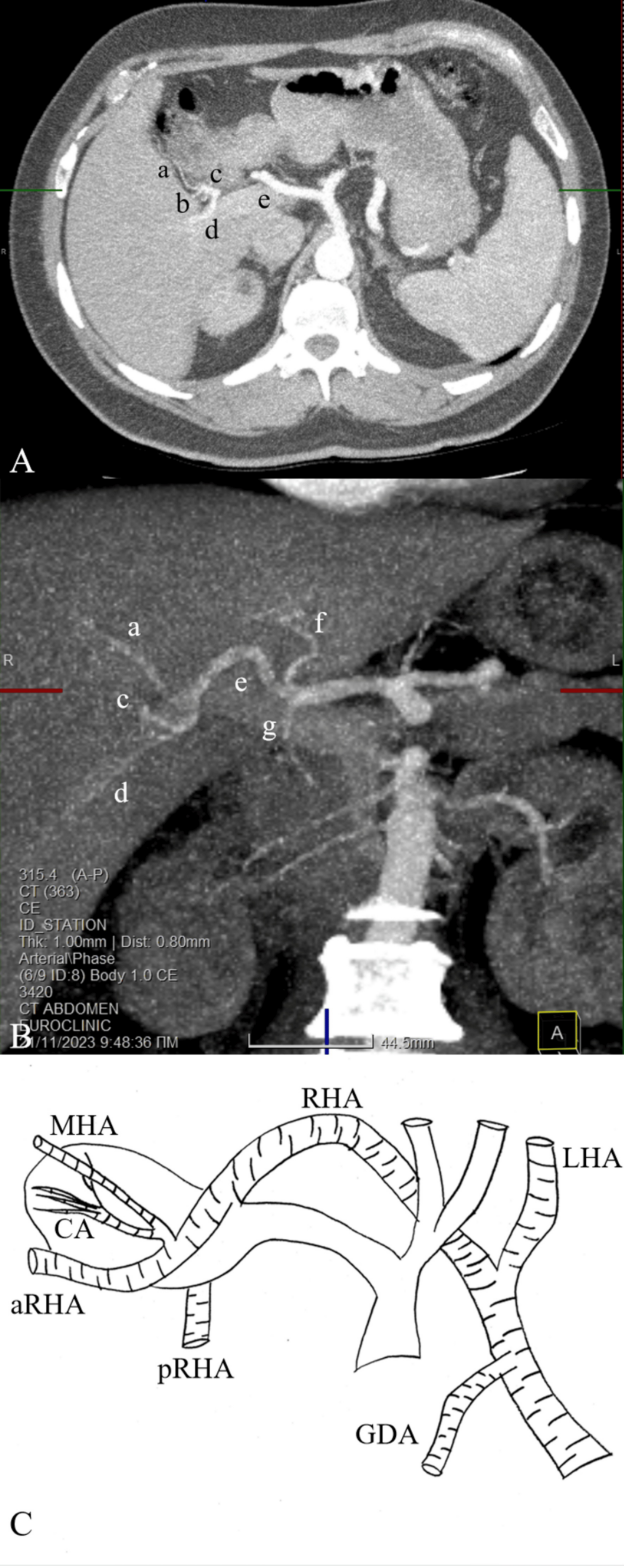
CT (A) coronal plane and (B) traverse plane, and (C) graphical image (a) Middle hepatic artery (ΜΗΑ), (b) cystic artery (CA) (clipped), (c) anterior branch of the right hepatic artery (aRHA), (d) posterior branch of the RHA (pRHA), (e) RHA, (f) left hepatic artery (LHA), (g) gastroduodenal artery (GDA) Image credit: Miltiadis Perdikakis

A CT scan was postoperatively conducted for further clarification of the hepatic arterial supply and depicted the origin of the arteries identified during the operation (Figure 2).

## Discussion

**Figure 3:**
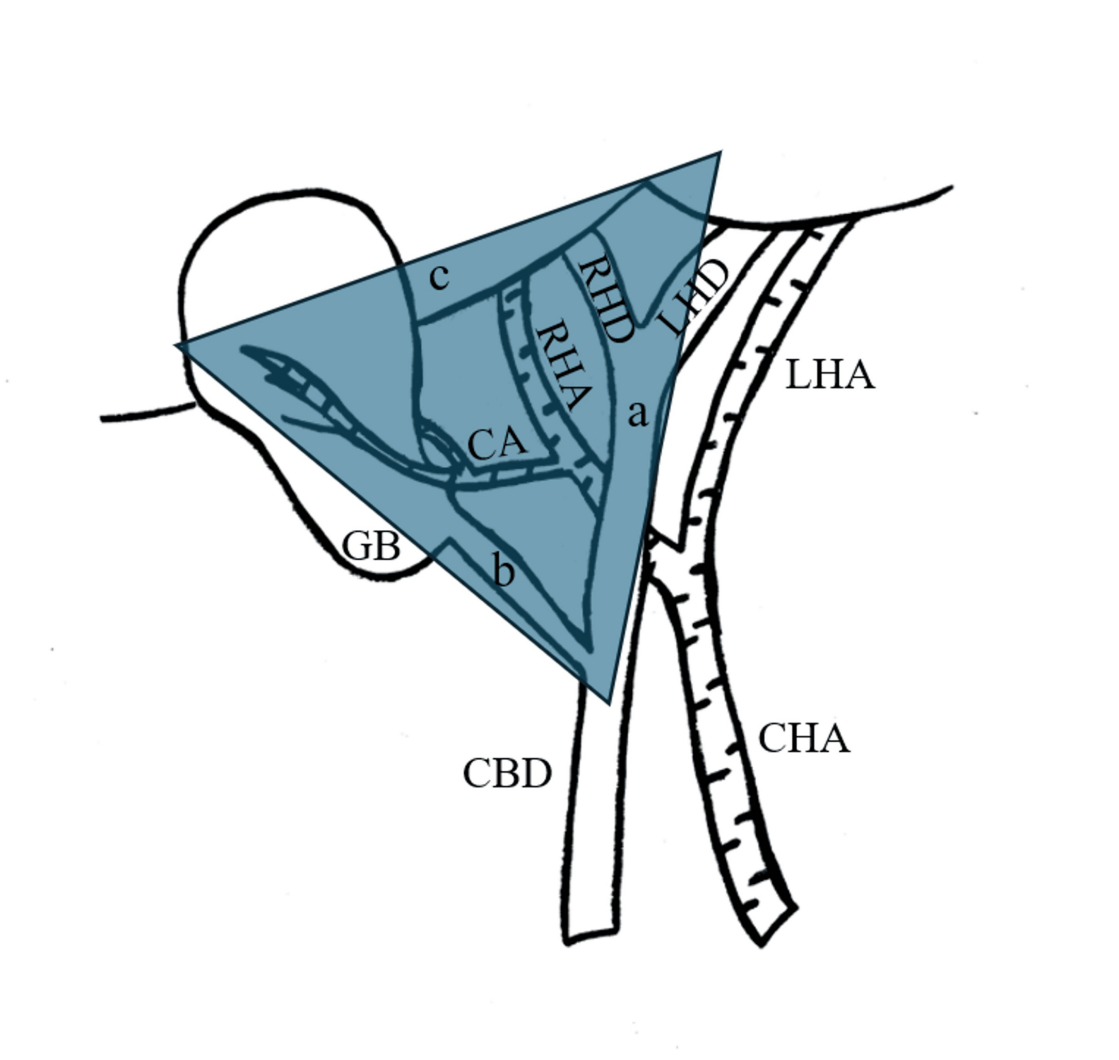
Calot's triangle (colored), as defined by the (a) common hepatic duct, (b) cystic duct, and (c) inferior surface of the liver LHA, left hepatic artery; RHA, right hepatic artery; CA, cystic artery; CHA, common hepatic artery; LHD, left hepatic duct; RHD, right hepatic duct; CBD, common bile duct; GB, gallbladder Image credit: Miltiadis Perdikakis

Laparoscopic cholecystectomy is the gold standard operation for gallbladder removal and has become a common surgical procedure; however, it is not without risks. The various anatomical structures that traverse Calot’s triangle (defined by the cystic duct, the common hepatic duct, and the inferior surface of the liver) may differ substantially from patient to patient [5]. This is particularly evident with the blood supply of the gallbladder. The cystic artery typically originates from the right hepatic artery, traverses Calot’s triangle and enters the gallbladder at the junction of its neck with the cystic duct [6]. As it approaches the gallbladder, it divides into two branches: the anterior superficial and the posterior deep. The former courses to the peritoneal surface of the gallbladder, while the latter runs between the gallbladder and its fossa (Figure 3) [7].

Aside from the typical anatomy, numerous arterial variations have been identified. The “caterpillar hump” is a characteristic example, which describes a long RHA that can be mistaken for the CA, potentially leading to a false ligation of the RHA [8]. Moreover, both the RHA and the CA can course anterior to the common bile and/or hepatic duct. Additionally, accessory CAs have been detected, whose bleeding may pose challenges during the operation. It is noteworthy that the anatomic variants of the arteries are more common than those of the ducts [9].

**Figure 4:**
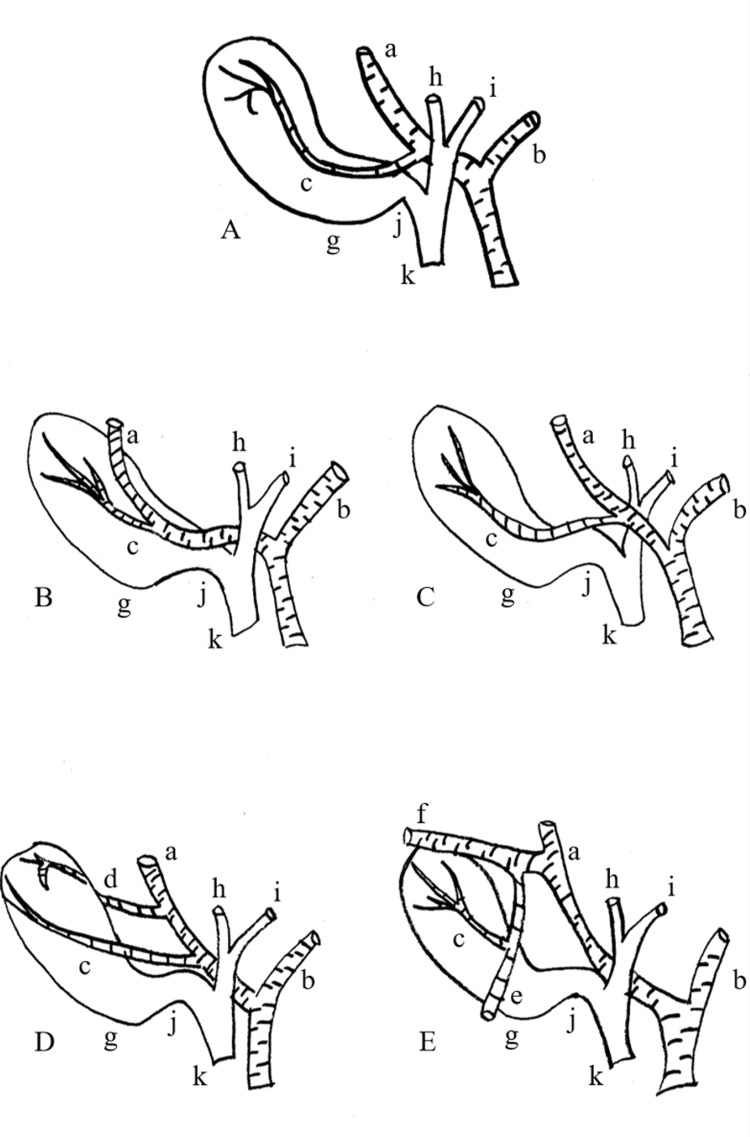
(A) Typical anatomy, (B) “caterpillar’s hump”, (C) right and left hepatic artery, anterior to the common bile and hepatic duct, (D) accessory cystic artery, (E) cystic artery from the middle hepatic artery (a) Right hepatic artery, (b) left hepatic artery, (c) cystic artery, (d) accessory cystic artery, (e) middle hepatic artery, (f) anterior branch of the right hepatic artery, (g) gallbladder, (h) right hepatic duct, (i) left hepatic duct, (j) cystic duct, (k) common bile duct Image credit: Miltiadis Perdikakis

The current case is even more uncommon than those mentioned above. The CA, in this instance, is a branch of the MHA, which supplies the IV hepatic segment and closely resembles the typical CA (Figure 4).

In such cases, there is a significant risk of mistakenly ligating the MHA and compromising the hepatic blood supply. The precise origin of the MHA varies among a number of studies. It mainly arises either from the LHA or the RHA and seldom from both (dual MHA) or from the proper hepatic artery [10,11]. In our case, the MHA originated from the anterior branch of the RHA, a variation rarely described [12]. Regarding the CA, in less than 5% of cases it is a branch of the MHA, as indicated in a study [13]. It is noteworthy that in a review of CA variations based on numerous cases, no instances of the CA branching from the MHA were identified [2]. In our PubMed search, only two similar case reports were found [14,15]. In our case, the anatomy was even more complex, since two rare sights, CA arising from the MHA and MHA arising from the anterior branch of the RHA, emerged concurrently.

Ligation of the MHA does not bibliographically present major compilations. It could sometimes cause complications such as thrombosis, ischemic cholangiopathy or reduction of the left hepatic lobe [16]. The usual elevation of the hepatic enzymes following cholecystectomy would be greater, even though it is not a valid indicator of the hepatic function and postoperative complications [17]. Therefore, unnecessary medical interventions and prolonged hospitalization could hinder the patient’s discharge. However, in cases like the present one, in which the MHA originates from the anterior branch of the RHA, segment IV’s ischemia might be unavoidable if the MHA is ligated [8].

The main method of performing a safer cholecystectomy is achieving the critical view of safety. It consists of three main steps. First, the Calot’s triangle is dissected; then, it is important that the lower third of the gallbladder is exposed, so that the possible structures of the area are exhibited. Finally, the cystic duct and the CA are identified, as they are the only tubular vessels still attached to the gallbladder [18]. This method provides a better overview of the structures that are to be ligated and removed together with it, preventing the ligation of false components. Another useful approach for more complex operations, when the structures cannot be easily distinguished, is the retrograde cholecystectomy, in which the dissection begins from the gallbladder’s fundus and then approaches the infundibulum [19]. In this way, the structures that are running towards the gallbladder can be easily identified.

## Conclusions

The laparoscopic anatomy during cholecystectomy can differ from the classical anatomy found in the medical handbooks. Therefore, the surgeon should always be aware of encountering a rare sight, with anatomic variations. Pre-operative ultrasound for imaging of possible gallstones cannot detect these variations. Thus, the peri-operative identification of the anatomical structures is of utmost importance. The careful dissection of the Calot’s triangle and the achievement of the critical view of safety should always be important for all surgeons. In certain cases, the retrograde cholecystectomy could help restore the correct orientation and might prove to be a useful approach in unclear anatomy. This case report presents a rare anatomical variation in which the cystic artery originated from the middle hepatic artery, a phenomenon that, to our knowledge, has been scarcely described in the existing literature and could possibly challenge surgical procedures.
